# Nighttime Continuous Contactless Smartphone-Based Cough Monitoring for the Ward: Validation Study

**DOI:** 10.2196/38439

**Published:** 2023-02-20

**Authors:** Filipe Barata, David Cleres, Peter Tinschert, Chen-Hsuan Iris Shih, Frank Rassouli, Maximilian Boesch, Martin Brutsche, Elgar Fleisch

**Affiliations:** 1 Center for Digital Health Interventions Department of Management, Technology, and Economics ETH Zurich Zurich Switzerland; 2 Resmonics AG Zurich Switzerland; 3 Lung Center Cantonal Hospital St. Gallen St. Gallen Switzerland; 4 Center for Digital Health Interventions Institute of Technology Management University of St. Gallen St. Gallen Switzerland

**Keywords:** cough monitoring, ward monitoring, mobile sensing, machine learning, convolutional neural network, COVID-19, mobile phone

## Abstract

**Background:**

Clinical deterioration can go unnoticed in hospital wards for hours. Mobile technologies such as wearables and smartphones enable automated, continuous, noninvasive ward monitoring and allow the detection of subtle changes in vital signs. Cough can be effectively monitored through mobile technologies in the ward, as it is not only a symptom of prevalent respiratory diseases such as asthma, lung cancer, and COVID-19 but also a predictor of acute health deterioration. In past decades, many efforts have been made to develop an automatic cough counting tool. To date, however, there is neither a standardized, sufficiently validated method nor a scalable cough monitor that can be deployed on a consumer-centric device that reports cough counts continuously. These shortcomings limit the tracking of coughing and, consequently, hinder the monitoring of disease progression in prevalent respiratory diseases such as asthma, chronic obstructive pulmonary disease, and COVID-19 in the ward.

**Objective:**

This exploratory study involved the validation of an automated smartphone-based monitoring system for continuous cough counting in 2 different modes in the ward. Unlike previous studies that focused on evaluating cough detection models on unseen data, the focus of this work is to validate a holistic smartphone-based cough detection system operating in near real time.

**Methods:**

Automated cough counts were measured consistently on devices and on computers and compared with cough and noncough sounds counted manually over 8-hour long nocturnal recordings in 9 patients with pneumonia in the ward. The proposed cough detection system consists primarily of an Android app running on a smartphone that detects coughs and records sounds and secondarily of a backend that continuously receives the cough detection information and displays the hourly cough counts. Cough detection is based on an ensemble convolutional neural network developed and trained on asthmatic cough data.

**Results:**

In this validation study, a total of 72 hours of recordings from 9 participants with pneumonia, 4 of whom were infected with SARS-CoV-2, were analyzed. All the recordings were subjected to manual analysis by 2 blinded raters. The proposed system yielded a sensitivity and specificity of 72% and 99% on the device and 82% and 99% on the computer, respectively, for detecting coughs. The mean differences between the automated and human rater cough counts were −1.0 (95% CI −12.3 to 10.2) and −0.9 (95% CI −6.5 to 4.8) coughs per hour within subject for the on-device and on-computer modes, respectively.

**Conclusions:**

The proposed system thus represents a smartphone cough counter that can be used for continuous hourly assessment of cough frequency in the ward.

## Introduction

### Background

Patient monitoring is the repeated or continuous observation of vital signs or physiological functions to ensure patient safety and guide therapeutic interventions [1]. Most modern cardiorespiratory monitoring systems rely on invasive sensors, cables, and bulky monitors to detect, transmit, process, and display the biosignals to be monitored [1]. Although most of these advanced monitoring systems are performed in the intensive care unit, nearly half of all adverse events in patients admitted to hospitals occur in the general care ward [2,3]. In fact, patients often die in wards where clinical deterioration can go unnoticed for hours [4]. For example, in the United States, acute respiratory events in inpatient wards are associated with in-hospital mortality of approximately 40% [5]. In the European Surgical Outcomes Study [6], which included 46,539 patients from 498 hospitals in 28 countries, most (73%) patients who died had not been admitted to the intensive care unit at any time after surgery.

Current monitoring protocols in the wards usually consist of random checks by a nurse approximately every 4-8 hours [1]. This leaves patients unattended most of the time during their hospital stay [7]. Changes in vital signs as warning signs of clinical deterioration are often not detected or are detected too late in conventional assessments during random checks. A closed claims analysis of opioid-induced respiratory problems in a general care ward found that nearly half of all health adverse events occurred within 2 hours of the last nursing check [8]. Furthermore, the authors concluded that almost all of these events could have been prevented with better ongoing monitoring and education [8]. Not only are critical changes in vital signs missed but also the detection of abnormal vital signs by a bedside nurse often triggers a long chain of commands that results in delays until action can be taken [9]. With the widespread adoption of mobile technologies such as smartphones and wearables, researchers have recognized that these technologies can facilitate continuous monitoring and may improve patient outcomes in hospital wards [4,10]. Coughing, in particular, holds great potential for monitoring through mobile technologies in the ward. Coughing is one of the most common medical complaints [11-13]. It is known as a symptom of the common cold. Nevertheless, it is associated with many prevalent communicable and noncommunicable respiratory diseases, asthma, lung cancer, and lower respiratory tract infections, including COVID-19, as well as with 2 of the top 10 causes of death worldwide, chronic obstructive pulmonary disease and tuberculosis [14]. Cough is not only a symptom but also a predictor of acute adverse health events. It is associated with exacerbations, lung function decline, and risk of death [15-17]. In addition, cough detection approaches involving smartphones and wearable recordings have been proposed and developed [18-20]. Overall, the results provide a proof of principle for cough detection with different devices in various settings. To date, however, there is no standardized method for cough quantification, and there is no adequately validated generic cough monitor that is commercially available, let alone clinically acceptable [12]. In fact, only a few of these systems have been validated in independent studies with different cohorts [21-27] (Table 1). The Leicester Cough Monitor is among the best evaluated ones, and 2 × 1-hour and 6-hour cross-sectional recordings of patients with chronic cough were used for validation [22]. Consequently, most methods and approaches developed only include the validation of the algorithm on audio recordings, meaning they do not include the validation on the device that has been designed for a real-world scenario. In addition, it has not yet been demonstrated that a smartphone can continuously monitor cough counts (in the ward).

Considering that most random checks of vital signs by nurses have gaps of approximately 4 hours between 2 consecutive assessments and that this period is associated with the highest risk, continuous accessible hourly cough monitoring would be desirable. Furthermore, these shortcomings in validation also limit the adoption of smartphones for *patient* monitoring of coughing and, consequently, hinder the potential that such scalable technologies could bring for the assessment of progression of prevalent diseases in the ward. To this end, this study proposes a smartphone-based cough monitoring system for the ward. Our approach differs from that used in previous research because our key innovation is to use smartphones to record and detect coughs in a contact-free and continuous manner in the ward. The number of detected coughs and their time stamps are transferred continuously to a server and displayed on a web client, whereas the corresponding recorded *data* are saved locally. The monitoring system is especially suited to monitor patients who are hospitalized with an aggravated condition. It operates contact free with low burden and minimal obtrusion for the *patient* and provides remote continuous cough monitoring, which the medical personnel can access.

**Table 1:** Overview of prior automatic and semiautomatic cough counting tools.

Cough monitor			Hardware		Recording modality		Automation		Cough quantification		Contact free		Real-time monitoring		Validation study (participants, n)		Reported performance in validation study									Comments
																	TPR^a^		TNR^b^		FP^c^		DIFF^d^			
Cayetano CoughMonitor [27,28]			Marantz PMD620 handheld recorder		Free-field microphone		Partial		Episode		No		No		Yes (—^e^)		75.5%		99.6%		Median 4 *ch*^−1f^		—		Results reported on a total of 49x30-minute-long MP3 recordings of patients with tuberculosis; sensitivity for single coughs: 51.4%	
LEOSound lung sound monitor [29]			Custom-built device		3 contact microphones		Full		Single		No		No		Yes (48)		98.7%		80.2%		—		—		Results reported on nocturnal recordings of maximum 10 hours in patients with COPD^g^; published validation study [26] did not undergo peer review	
LifeShirt [24]			Custom-built device		Plethysmography, EMG^h^, and electrocardiogram		Full		Single		No		No		Yes (8)		78.1%		99.6%		—		—		Results reported on a maximum 24 hours per patient and a total of 109 hours of recording in patients with COPD	
LR102 [25]			Custom-built device		3 EMG sensors and a contact sound transducer		Full		Single		No		No		Yes (10)		—		—		—		Mean −12.5 *ch*^−1^		Results reported on a total of 40 hours of recordings of patients with cystic fibrosis or a viral infection	
PulmoTrack-CC [23]			Custom-built device		2 contact microphones and a pneumogram belt		Full		Single		No		No		Yes (10)		26%		—		—		Median −100 *c* (4 *h*)^−1^		Results reported by the independent evaluation by Turner et al [30] on 4-hour-long hour recordings per participant with different conditions; original paper reported results on voluntary coughs [23]	
The Hull Automatic Cough Monitor [31]			Sony TCD-D8 Walkman DAT^i^ recorder		Free-field microphone		Partial		Single		No		No		No (10)		80%		96%		—		Mean 10 *ch*^−1^		Results reported on 1-hour-long recordings of a disjoint set of 10 participants (ie, smokers experiencing chronic cough) who were part of the same data collection study used to train the algorithm	
The Leicester Cough Monitor [22]			Archos Jukebox Recorder 20		Free-field microphone		Partial		Single		No		No		Yes (9)		91%		99%		Mean 2.5 *cp*^−1^*h*^−1j^		Mean −4 *cp*^−1^*h*^−1^		Results reported on 2×1-hour-long recordings of each patient with chronic cough [22]; data annotated by 2 raters	
The Leicester Cough Monitor [22]			Archos Jukebox Recorder 20		Free-field microphone		Partial		Single		No		No		Yes (23)		86%		99%		Mean 1.0 *cp*^−1^*h*^−1^		—		Results reported on 6-hour-long recordings of each patient with chronic cough [22]; data annotated by 1 rater	
VitaloJAK [21]			Custom-built device		Free-field microphone and contact microphone		Partial		Time		No		No		Yes (10)		99.92%		—		—		—		Results reported on 24-hour recordings on patients with different conditions; authors evaluated the system by confirming that the identified cough sounds in the compressed files are the same sounds identified by the trained manual cough counters in the full 24-hour recordings; algorithm compresses 24-hour-long recordings to an average of 26.30 minutes, which requires manual counting	
**Proposed system**																										
	Proposed system on computer	Smartphone (Samsung Galaxy A3); Python; Tensorflow		In-built microphone		Full		Single		Yes		No		Yes (9)		82%		99%		Mean 0.6 *cp*^−1^*h*^−1^		Mean −0.9 *cp*^−1^*h*^−1^		Results reported on 8-hourlong recordings per patient during the night (11 PM-7 AM) and a total of 72 hours of recordings in patients with pneumonia		
	Proposed system on device	Smartphone (Samsung Galaxy A3)		In-built microphone		Full		Single		Yes		Yes		Yes (9)		71%		99%		Mean 1.2 *cp*^−1^*h*^−1^		Mean −1.2 *cp*^−1^*h*^−1^		Results reported on 8-hour-long recordings per patient during the night (11 PM-7 AM) and a total of 72 hours of recordings in patients with pneumonia		

^a^TPR: true positive rate.

^b^TNR: true negative rate.

^c^FP: false positive rate.

^d^DIFF: difference between automated and annotated cough counts.

^e^Not available.

^f^*ch^-1^*: coughs or cough episodes per hour (see the *Quantificationof Cough* section).

^g^COPD: chronic obstructive pulmonary disease.

^h^EMG: electromyography.

^i^DAT: digital audio tape.

^j^*cp^-1^h^-1^*: coughs or cough episodes per patient per hour (see the *Quantificationof Cough* section).

### Objective

In this work, we contribute to existing research through the following contributions. First, to the best of the authors’ knowledge, this work is the first validation of a holistic smartphone-based cough detection system applied in a real-life scenario and validated with a different cohort compared with the one used to develop the models. Second, the proposed system not only encompasses cough detection but also comes with 2 additional functionalities, recording and continuous transmission of cough detection in near real time. Recording, on the one hand, allows for verifying the results and closing the loops on detection errors. Continuous transmission, on the other hand, enables the application of the technology in a remote monitoring setting and the visualization of cough counts in near real time. Third, we demonstrate the applicability of the proposed system in the ward for patients with an acute respiratory condition, that is, pneumonia. Moreover, by using consumer-centric devices such as smartphones, which are increasingly available in low- and middle-income countries, our proposed system allows for a scalable and cost-effective ward monitoring tool that can be used in low- and middle-income countries.

This work builds upon our prior research [20,32], in which we developed cough detection models based on coughs from patients with asthma. We evaluated the proposed system with 9 patients hospitalized for lower respiratory tract infections (3 infected with SARS-CoV-2) at night (ie, 11 PM-7 AM).

## Methods

### Study Design

This validation study compares automated cough counts with those identified using manual sound analysis. For this purpose, we targeted an adult patient population (aged 18 years) hospitalized for lower respiratory tract infection at the Lung Center of the Cantonal Hospital St Gallen, Switzerland. This study was motivated by the emergence of the COVID-19 pandemic and was designed, planned, and conducted in March 2020 and April 2020.

Patients enrolled in the study underwent nocturnal (ie, 11 PM-7 AM) cough monitoring using a contact-free smartphone-based cough detection system. To conduct the study, we used smartphones (Samsung Galaxy A3 2017, SM-A320FL, 2 GB RAM, Exynos 7870 Octa: 14 nm, Octa-core 1.6 GHz Cortex-A53, Wi-Fi 802.11a/b/g/n/ac) with the study app installed and equipped with secure digital cards (SanDisk Ultra microSDXC A1 64 GB 100MBs Adapt) to expand the memory for audio recording.

**Figure 1:**
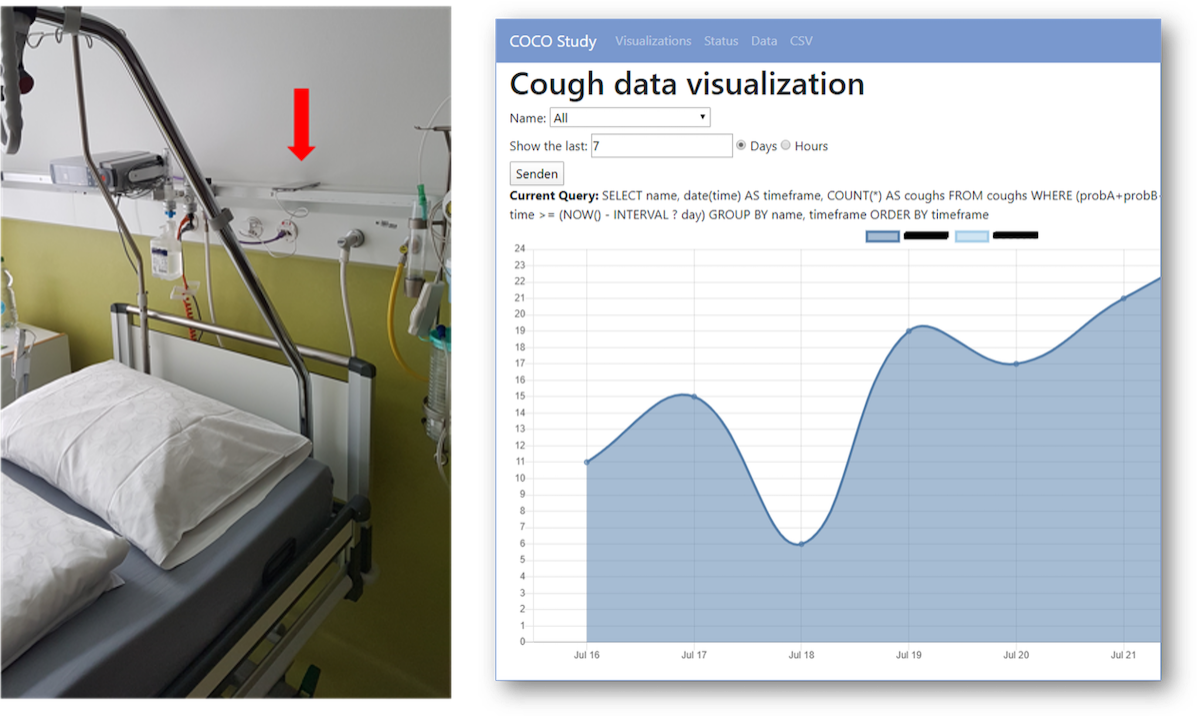
Experimental setup of the study (left) and cough count visualization on the web server (right). The experimental setup shows where the smartphones were placed and where the bed was located in the hospital room. The cough count visualization shows the cough counts per night of a patient. COCO: COugh in COvid-19; CSV: comma-separated values.

We placed the smartphones above the bed after the patients were admitted to the study in the hospital and had provided consent to be recorded. In addition, during the study, we used a web server to monitor cough counts in near real time, visualize them, and ensure that the technology was working (*Cough Detection* section). Figure 1 shows the experimental setup and a screenshot of the web server displaying the cough counts. Of the total nights of hospital stay of each patient, we randomly selected 1 night (ie, 8 hours) for validation purposes. Two trained annotators labeled the selected recordings according to a predefined annotation protocol.

### Quantification of Cough

For quantification purposes, the definition of cough depends on the signals used for the measurement. Most commonly, cough is counted based on sounds, either alone or in combination with a second signal [12]. Quantification of coughs based on sound recordings can be accomplished using different methods. Counting the characteristic explosive cough sounds is the most commonly used metric for quantifying cough and is used in this study [12]. The counting of explosive cough sounds is sometimes referred to as *cough frequency* in the literature.

### Data Annotation

For the annotation process, we used the same labeling manual that we published in our previous study [20]. First, annotators marked silence by applying a decibel filter to the recordings using the Audacity software (The Audacity Team). The Sound Finder filter marked sounds <−26 dB as silence with the restriction that the minimum duration of silence between sounds was 1 second. These periods marked as silence served as visual aids for the rest of the annotation process. Human annotators listened to the smartphone recordings and marked the periods not marked as silence as coughs when an explosive cough sound was identified [12,33].

We used 2 approaches to guarantee the quality of labeling. First, we instructed human annotators to label an acoustic event if they were unsure whether it was a cough. If the annotators were unsure, the event was discarded and excluded from the analysis. The remaining acoustic events were classified as noncough. Second, to assess the quality of the annotations and determine interrater reliability, we used the intraclass correlation coefficient.

### Ethics Approval

The study protocol was reviewed and approved by Ethikkommission Ostschweiz, which is responsible for research on humans in Eastern Switzerland (Business Management System for Ethics Committees ID BASEC ID: 2020-00741).

### Proposed System

The proposed system consists primarily of an Android app running on a smartphone that detects coughs and records sounds and secondarily of a backend that continuously receives the cough detection information and shows the hourly cough counts. This gives rise to the two modes of use that are validated in this work: (1) near real-time smartphone-based cough monitoring through on-device cough detection and (2) a posteriori cough monitoring through on-device recording and on-computer detection.

### On-Device Cough Detection

**Figure 2:**
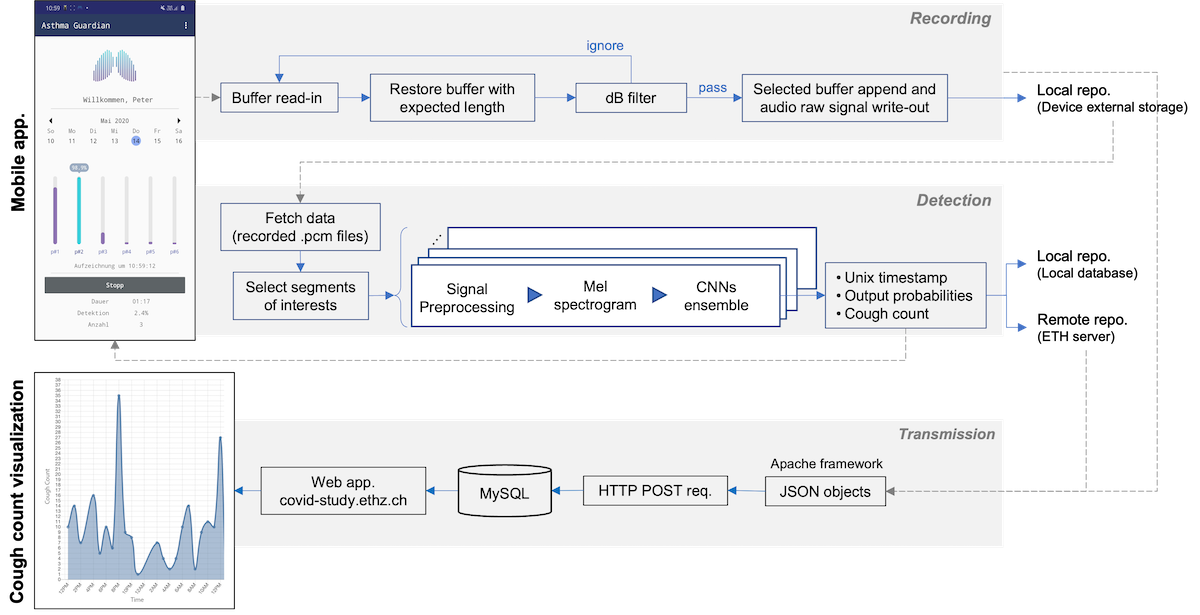
The proposed cough monitoring system. The figure shows the recording, the detection of cough on the running Android app, the transmission, and the visualization of the cough counts on the web client. CNN: convolutional neural network. dB: decibel; repo: repository; ETH: Eigenössische Technische Hochschule; req: request.

The on-device cough detection system consists of 3 building blocks: recording, detection, and transmission (Figure 2). To enable the described building blocks, we developed an Android app with a foreground service for continuous recording and detection processes. Recording and detection are triggered and run in 2 separate threads (ie, recording thread and detection thread) to handle the latencies between the audio sampling rate and detection speed. The monitoring system is triggered manually by a start and stop button click in the app.

The recording thread consists of a continuous loop reading the audio buffer and passing the dB filter until 6.5-second–long audio segments are gathered and stored on the external data storage of the smartphone. We implemented the dB filter to discard 0.65-second audio segments that do not contain absolute amplitudes higher than −26 dB. The recording of the audible audio files is optional. For validating the performance of the proposed system in this study, we recorded the audio files in Waveform Audio File Format on an inserted SD card at a sampling frequency of 16 kHz, 16 bits/sample, and pulse-code modulation codec.

Simultaneously, the detection thread loads the produced pulse-code modulation–encoded files. From each 6.5-second–long audio file, six 0.65-second–long segments were extracted for cough detection. These 6 windows were centered on the 6 maximum absolute amplitudes of the signal, spaced at least half a window apart in the audio file. Cough detection is based on an ensemble convolutional neural network (CNN) developed and trained on asthmatic cough data [20,32]. The detection step followed the procedure previously reported by Barata et al [20]. We first normalized the extracted minimum-maximum windows and multiplied them using a Hanning window. We then filtered the output with a Butterworth high-pass filter of order 5 and a cutoff frequency of 10 Hz to reduce the low-band noise and discontinuity effects. Finally, we computed Mel spectrograms with 80 bands, 112 samples between successive frames, and a 2048-point fast Fourier transform yielding an 80×128-sized matrix. We computed the Mel spectrograms using the Melspectrogram function of the *librosa* (version 0.9.2) Python package [34].

**Figure 3:**
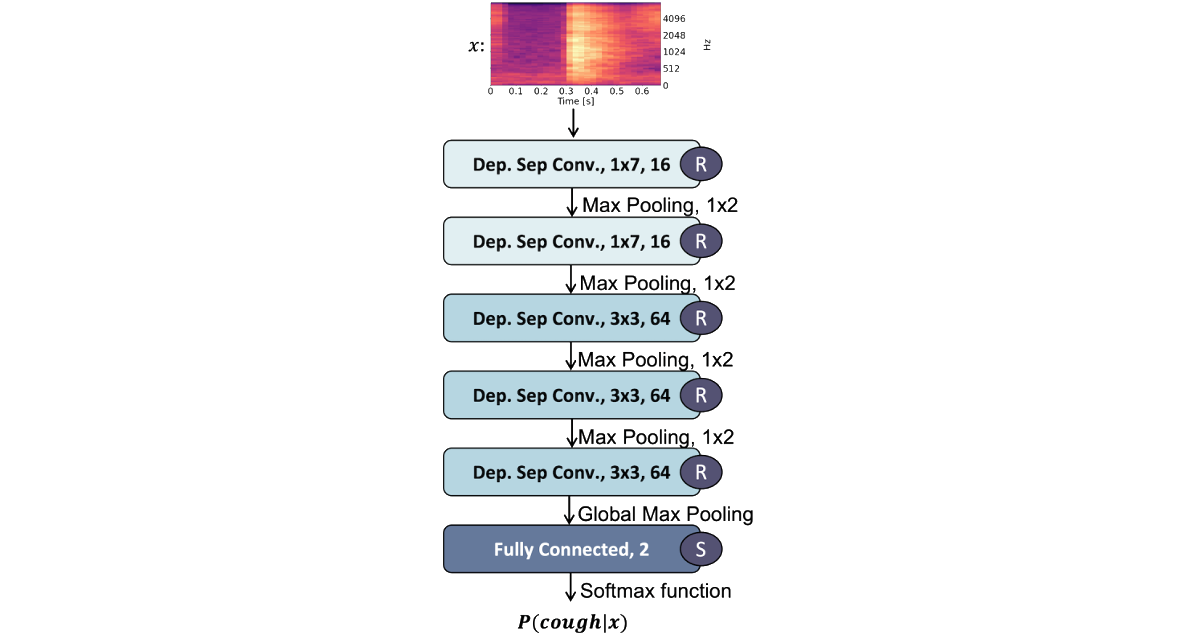
Convolutional neural network architecture. The annotations “Dep. Sep. Conv. 1 × 7, 16” refer to a depthwise separable convolutional layer with a 1 × 7 convolutional filter and 16 channels. “R” and “S” stand for the rectifying linear unit and the sigmoid activation function, respectively. P(cough∣x) refers to the predicted probability of the model. Such architecture represents one of the 5 models that make up the ensemble model.

Subsequently, these Mel spectrograms serve as input to the trained ensemble classifier, which outputs the cough probabilities. The trained ensemble classifier consists of 5 different CNN-based models. Each CNN-based models’ architecture consists of 5 convolutional layers with alternating max-pooling layers, followed by a global max-pooling layer. The architecture of a single CNN is illustrated in Figure 3. We computed cough probabilities as the average of the output cough probabilities of each CNN model. Subsequently, we sent the cough probabilities to a remote server. On the server, if the generated probability by the ensemble classifier was above a predefined threshold, a cough was counted. Alongside the cough probabilities, we encapsulated the time stamp when the cough event occurred (derived from the time of recording and the sampling rate), battery, and memory recorder statuses into JSON objects. These data objects are then sent in 2-minute intervals to the server by HTTP POST requests, stored in MySQL databases, and visualized using a web client (see the transmission box in Figure 2). In this way, the cough counts can be visualized on the web server within 2 to 3 minutes after the first recording on the device.

The monitoring app meets the following specifications, which were measured using Android Profiler, an integrated tool of Android Studio [35]. The Android app requires 90 MB for installation and Android 8 or a higher version. The RAM use of the monitoring app ranges between 133.9 MB when a recorded sound exceeds the threshold of the dB filter and 106.0 MB in the idle (eg, no noise) mode. The CPU use of the monitoring app ranges between 16% and 25% in idle and processing mode, respectively. The network use of the app consists of the HTTP POST requests for status updates (506 B/request) and information about the detected cough (1.6 kB/request). The Android Profiler estimates the battery consumption as “light,” which is reflected by an observed mean battery life of 12 hours and 1 minute (SD 1 hour and 19 minutes) while running the app. Fluctuations in the battery life can be attributed to the fluctuating noise during the recording.

The detection of cough in real-world data sets represents an imbalanced classification problem, that is, noncough sounds occur much more frequently than cough sounds. This raises the problem that a loss function on imbalanced data can easily be minimized by focusing on the majority class and overlooking the minority class. This problem can be alleviated by means of cost-sensitive learning, that is, by adopting a different loss function with different costs associated with each class [36]. Cost-sensitive learning can be applied during training and as a postprocessing step, introducing the cost factor when a decision regarding a new instance is being made [36].

This can be achieved by moving the decision threshold of the pretrained classifier. As derived by Fernandez et al [36], a new instance should be classified as one belonging to a class characterized by the lowest expected cost. The cost (C) can be considered as a penalty factor aiming at increasing the importance of the minority classes.

Hence, in a 2-class problem, a cost-sensitive classifier classifies a given instance *x* as belonging to the cough class if and only if

P (noncough∣x)⋅C_noncough_≤P(cough∣x)⋅C_cough_ (**1**)

From the fact that P (noncough∣x) = 1–P(cough∣x), a threshold *th* for classifying an instance *x* as belonging to a positive class (ie, cough) can be obtained if P(cough∣x)≥th, where



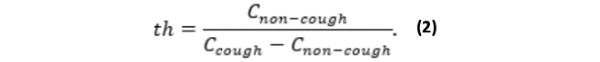



**Figure 4:**
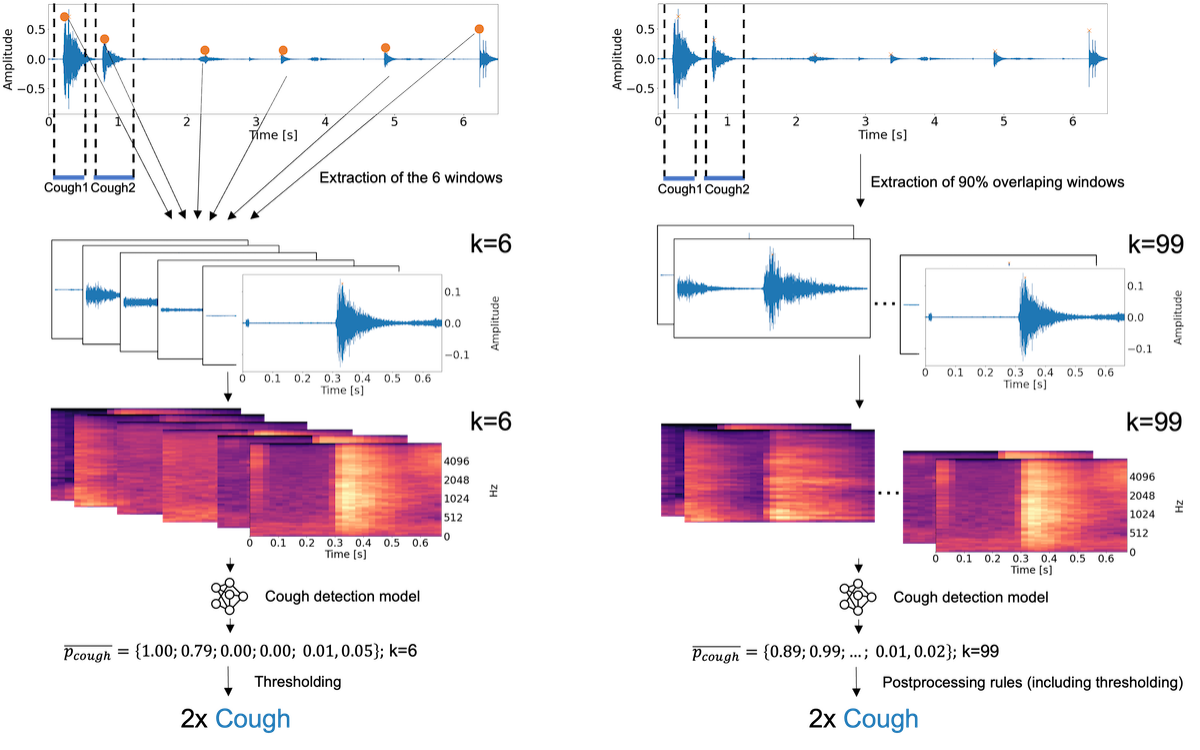
On-device (left) and on-computer (right) cough detection on 6.5-second-long audio file (from top to bottom): First, extraction of windows (extraction of 6 windows with maximal absolute amplitudes (left); continuous extraction of overlapping windows); second, the computation of Mel spectrograms; third, the computation of the prediction probability of cough by the convolutional neural network ensemble; last, segmentation of cough probabilities into cough counts (thresholding, left; postprocessing rules including thresholding). k= number of detections per file.

From equation (2), it follows that cost-sensitive learning can be used by moving the decision threshold of the pretrained model to convert it into a cough or noncough decision. Hence, we optimized the threshold in a previous work to adapt the detection model, which was trained on mostly well-controlled patients with asthma, that is, we used th_Asthma_=0.95 [20]. We determined the adapted threshold by maximizing the Matthews correlation coefficient (MCC) between the automated and annotated cough counts of patients with asthma [20]. The on-device cough detection algorithm is illustrated in Figure 4.

### On-Computer Cough Detection

The on-computer cough detection was conducted after the recording of the patient was complete and was executed on the recordings, which have been produced by the recording and filtering steps described in the *On-Device Cough Detection* section. It represents a similar mode of use as other commercially available solutions [21-25,27,29,31], which do not offer on-device analysis capability. It follows the algorithm described by Barata et al [20]. First, continuous overlapping windows from the continuous audio recordings were extracted. We extracted 650-millisecond-long windows with an overlap of 585 milliseconds. Second, Mel spectrograms were computed. Third, the prediction probability of cough was computed using the CNN ensemble. Finally, cough counts were generated by applying the following postprocessing rules: only consecutive probabilities above the predefined threshold were labeled as coughs to reduce the number of false detections; single probabilities above the threshold were then considered when the following probability was also above a second predefined threshold; and when >8 consecutive detections occur, 2 coughs were counted. Because the models were applied in a different context compared with the previous work, the thresholds were optimized to fit a different application context and differ from previous work [20], that is, we used the following thresholds: 0.66, 0.62. Figure 4 shows the application of the on-device cough detection algorithm in comparison with the on-computer cough detection algorithm. We found the adapted threshold by maximizing the MCC between the automated and annotated cough counts of the collected data set.

### Validation

In the validation, we compared automated cough counts with those identified by manual sound analysis in 8-hour-long recordings of patients with pneumonia. Manual analysis of sound recordings consisted of 2 blinded rater counts (rater1 and rater2). We compared the hourly cough counts generated through the proposed system with the annotated cough counts of one randomly selected rater. In this way, we can analyze the discrepancies between the proposed system and the rater. We argue that the selection bias of the results by choosing one of the raters can be neglected, provided that the 2 raters are in excellent agreement.

As performance metrics, we chose previously established metrics to compare a new measurement technique, such as the repeated measures correlation [37] and Bland-Altman plots [38]. Repeated measures correlation denoted by *r_m_* (with error degrees of freedom in parentheses) is a statistical technique for determining the common association within a patient for paired measures assessed on ≥2 occasions for multiple patients [37]. In addition, we used a scatter diagram to visualize the metrics [39]. The Bland-Altman plot analysis is a simple way to assess bias between mean differences and estimate an agreement interval into which 95% of the differences from the second method fall compared with the first method [40]. In our case, we wanted to evaluate the agreement between a continuous random variable *X* (eg, the proposed system cough counts) and a random variable *Y* (eg, human rater cough counts), both measuring the same underlying variable *D* within-individual, that is, cough counts per hour. Therefore, to compute the limits of agreement, we followed the procedure proposed by Zou [41] and modeled the difference between the counts measured by the proposed system and the rater for each pair of measurements as a one-way random effects model.

d_ij_ = x_ij–_y_ij_ = d + a_i_ + e_ij_ (**3**)

where *d* is an unknown true difference between the 2 methods, *a_i_* and *e_i_* are mutually independent normal variables with a mean of 0 and variances *σ^2^_b_* and *σ^2^_dw_*, respectively. The limits of agreement, in this case, are defined by the sum of the true difference *d*, random variability between (*a_i_*) and within-subject variability (*e_ij_*) Hence, the limits of agreement are as follows:

LoA_lower_ = μ_d_ – z_β\/2_ σ_d_ (**4**)

LoA_upper_ = μ_d_ + z_β\/2_ σ_d_ (**5**)

where *z_β/2_* is the upper *β/2* quantile of the standard normal distribution, which is usually set to 1.96. We computed the mean of the difference between methods for each pair of measurements per patient following the method described by Zou [41].

We further reported the average number of false positives per hour per patient and the average number of false negatives per hour per patient. To do so, we modeled the limits of agreement by the sum of true false positive or false negatives (*d*), the random variability between (*a_i_*) and within-subject variability (*e_ij_*) as in equations (4) and (5) and follow the same calculations as proposed by Zou [41]. We have also reported the classification metrics over all recordings, such as precision, recall (true positive rate [TPR]; also known as sensitivity), specificity (true negative rate [TNR]), negative predictive value (NPV), MCC, and cumulative distribution function of the error of the proposed system.

## Results

### User Statistics

A total of 10 participants with pneumonia (1/10, 10% female and 9/10, 90% males), 4 (40%) of whom were infected with SARS-CoV-2, were recruited for this validation study. The mean age of the participants was 66 (SD 11; range 52-85) years. None of the participants required ventilator support during the study. However, a ventilator was always available in case one was needed. We excluded 1 (10%) male participant from the analysis because the smartphone was erroneously placed (ie, not following the protocol; in direct proximity to the ventilator, masking patient sounds with constant background noise). We confirmed this observation empirically by randomly selecting twenty 1-second–long sequences of background noise for each participant and comparing the average energy of the signals per participant. We computed the energy of the signals by computing the power spectral density using Welch method [42] and integrating over the duration of the signal. The results yielded an energy of 777 for the excluded participants and 60, 104, 102, 92, 92, and 166 for the included participants. Hence, this test showed an approximately 5- to 8-fold increase in the background noise signal energy for the excluded participants compared with the other participants.

Consequently, we analyzed a total of 72 hours (ie, 9 × 8 h) of recordings. All recordings were manually analyzed by 2 blinded raters, hereafter referred to as rater1 and rater2. We calculated the intraclass correlation coefficient based on all nights, yielding a value of 98.8%. We interpret this value as excellent.

In the following section, we refer to the annotations made by rater1 as the reference for our analysis and compare the predictions made by the proposed system to the cough counts made by rater1. Cough counts per hour ranged from 0 to 33 coughs with a mean of 7.8 (SD 8.5) coughs.

### Evaluation Outcomes

For analyzing the same amount of data as the human raters, the on-device system produced 8298 predictions and the on-computer system produced 134,492 predictions. As shown in Table 2, the cough classification of the proposed on-device system yielded an MCC of 75%, a recall (TPR) of 71%, a specificity (TNR) of 99%, a precision (PPV) of 83%, and an NPV of 98%. The on-computer algorithm yielded an MCC of 86%, a TPR of 82%, a TNR of 99%, a PPV of 92%, and an NPV of 99%.

**Figure 5:**
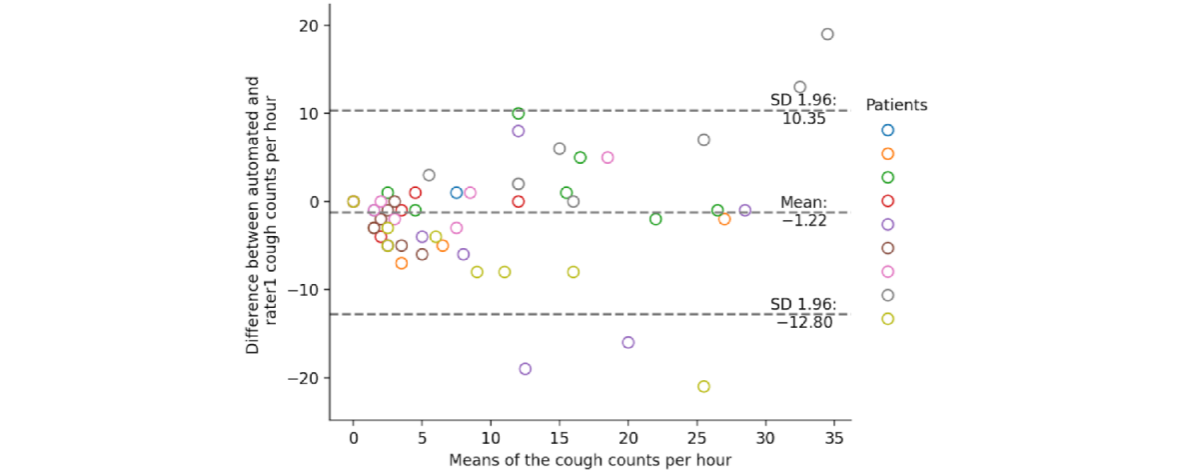
Bland-Altman plot of the rater1 and the on-device automated cough counts per hour.

**Figure 6:**
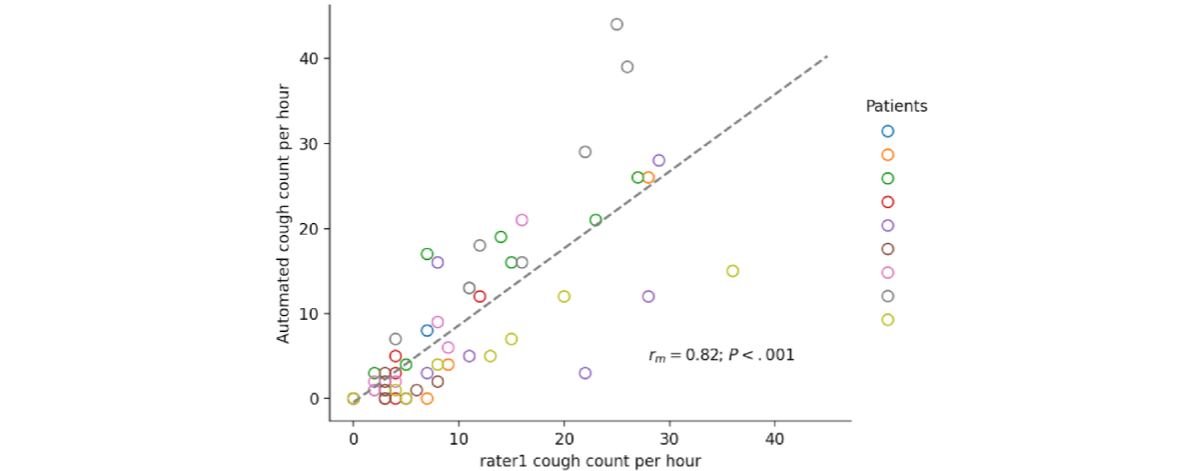
Correlation diagram of the rater1 and the on-device automated cough counts per hour.

The proposed on-device system and rater1 cough counts yielded a mean difference of −1.2 (95% CI −12.8 to 10.3) coughs per hour within subject and a within-subject correlation of *r_m_*_62_=0.82; *P*<.001; 95% CI 0.72 to 0.89. Figures 5 and 6 show the corresponding Bland-Altman plot and the correlation diagram between the hourly cough counts of the proposed system and rater1, respectively. Furthermore, the proposed system achieved a mean false positive rate of 1.2 (SD 3.31; 95% CI 0.0-7.7) coughs per hour within subject and a mean false negative rate of 2.4 (SD 4.22; 95% CI 0.0-10.7) coughs per hour within subject.

**Figure 7:**
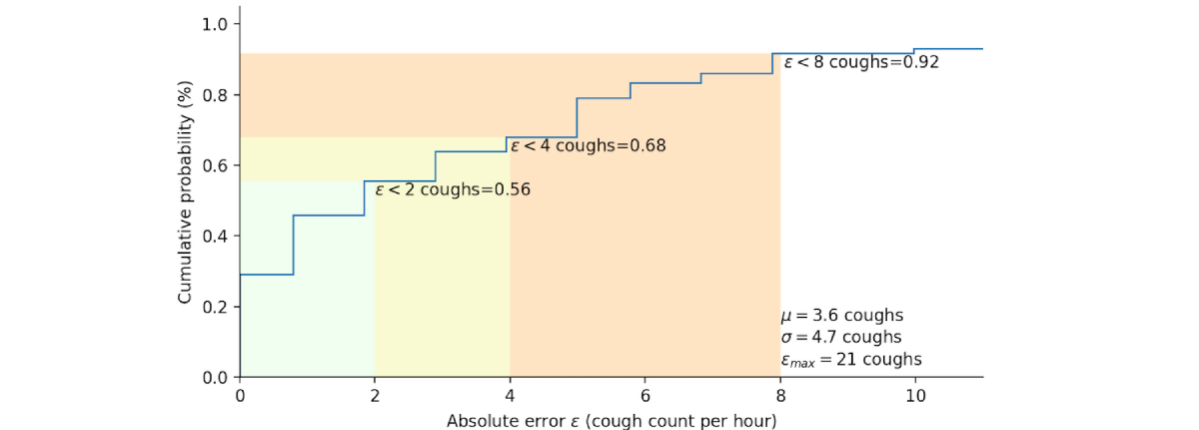
Cumulative probability distribution plot of the absolute error between rater1 and the on-device automated cough counts per hour.

Finally, as shown in Figure 7 for the cumulative distribution function, 56% of the absolute errors fall under 2 coughs; 68% fall under 4 coughs; and 92% fall under 8 coughs, with a maximum of 21 coughs for the on-device cough monitoring system. In total, rater1 counted 594 coughs. The proposed system counted a total of 506 coughs.

**Figure 8:**
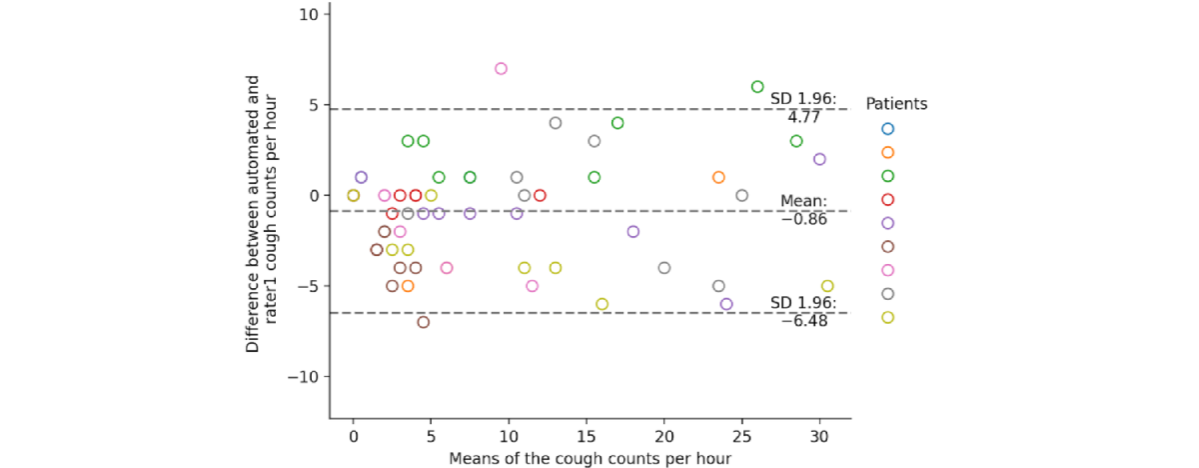
Bland-Altman plot of the rater1 and the on-computer automated cough counts per hour.

**Figure 9:**
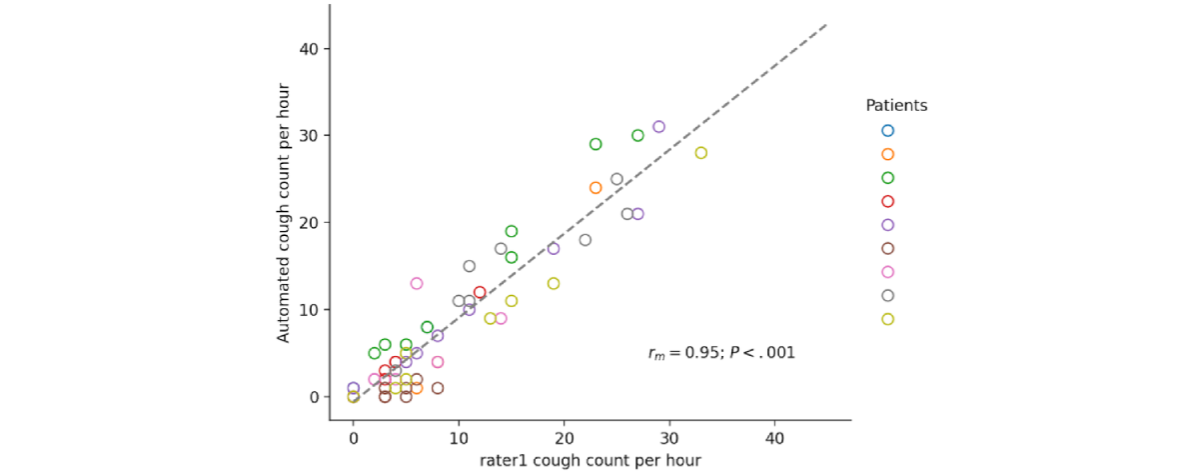
Correlation diagram of the rater1 and the on-computer automated cough counts per hour.

The on-computer and rater1 cough counts yielded a mean difference of −0.9 (95% CI 95% −6.5 to 4.8) coughs per hour within subjects and a within-subject correlation of *r_m_*_57_=0.95; *P*<.001; 95% CI 0.92-0.97. Figures 8 and 9 show the corresponding Bland-Altman plot and the correlation diagram between the hourly cough counts of the on-computer system and rater1.

**Figure 10:**
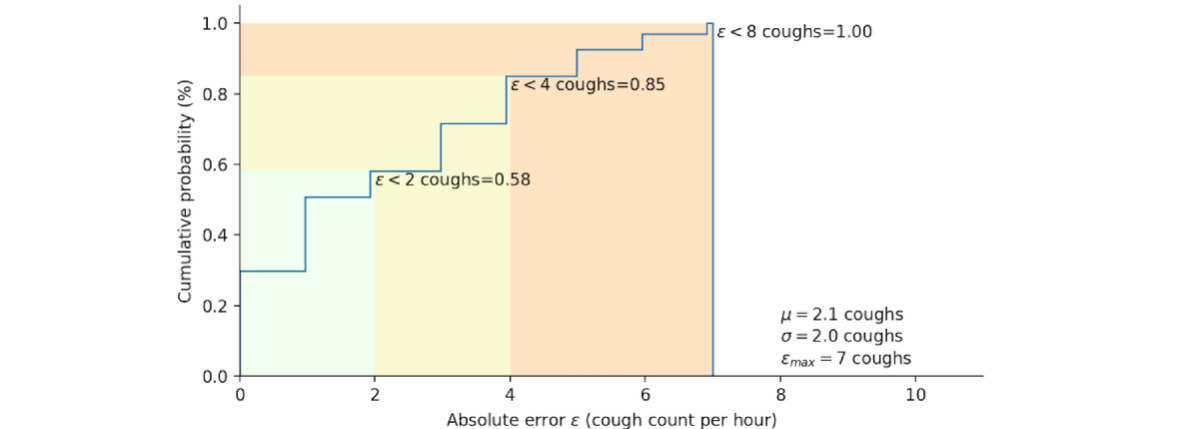
Cumulative probability distribution plot of the absolute error between rater1 and the on-computer automated cough counts per hour.

Furthermore, the on-computer mode achieved a mean false positive rate of 0.6 (SD 1.45; 95% CI 0.0-3.5) coughs per hour within subjects and a mean false negative rate of 1.46 (SD 2.05; 95% CI 0.0-5.5) coughs per hour within subjects. In the on-computer system, 58% of absolute errors fall under 2 coughs; 85% fall under 4 coughs; and 100% fall under 8 coughs, with a maximum of 7 coughs (Figure 10).

**Table 2:** Classification performance over all recordings.

	TPR^a^	PPV^b^	TNR^c^	NPV^d^	MCC^e^
On device	71%	83%	99%	98%	75%
On computer	82%	92%	99%	99%	86%

^a^TPR: true positive rate.

^b^PPV: positive predictive value.

^c^TNR: true negative rate.

^d^NPV: negative predictive value.

^e^MCC: Matthews correlation coefficient.

## Discussion

### Principal Findings

From the results discussed in the *Evaluation Outcomes* section, we conclude that the 2 modes of the proposed system can detect nocturnal cough counts per hour that correlate strongly with human raters in a clinical setting. In particular, the on-computer monitoring system yielded a repeated measures correlation and mean cough count differences close to those encountered in the analysis of measurements by human raters. The on-device system performed worse in terms of recall yet demonstrated a strong correlation between automated and human rater hourly cough counts, with the advantage that it was notably more efficient. The on-device cough monitoring system required only about 5% of the number of predictions needed to perform cough detection on all recordings compared with the on-computer cough monitoring system. Although both modes of the proposed system do not reach the levels of agreement of human raters, it must be emphasized that this is not a fair comparison, as this does not constitute the standard that a human can reproduce under real conditions with her or his abilities. Rather, by “human raters,” we mean computer-aided, trained human raters who had the advantage of being able to mark silence in the recordings using Audacity software, the ability to save their current work status, and the ability to pause at will during the annotation process. The discrepancy in performance between the 2 systems can be best explained by first, the number of predictions, which is limited to 6 predictions per 6 seconds for the on-device system and limits the ability to recognize true positives, and second, the postprocessing rules on the on-computer system, which can eliminate single false positives. We explain the drop in recall in comparison with our findings in previous work [20] not only by the differences in the algorithm but also by the cough type and frequency. Because the CNN-based classifier was trained on data from patients with asthma and recorded at home, most of whom lived under controlled conditions, in this study, we applied the classifier to older patients who were hospitalized because of a lower respiratory tract infection. We also selectively listened to the original recordings and believe that the differences in the automated and human rater cough counts stem from errors caused by strong background noise, throat clearing, and strong abrupt breath sounds among others; improving the model is certainly indicated in such cases. With its dual functionality of recording and detection, the proposed system identifies these noises and provides a systematic approach for closing the gap in detection errors. In addition, our system successfully enabled continuous monitoring of the patient by medical staff through the continuous transmission of cough counts and visualization on the web server (Figure 1).

### Limitations

There are limitations regarding the generalization of our results. First, limited time was available for optimizing the on-device processing pipeline because the system was deployed only a month after starting the first COVID-19–related lockdown. Hence, the results of the on-device system underestimated the actual potential of the CNNs developed and trained by Barata et al [20] for real-time and on-device detection of cough. Second, we used only a specific smartphone model in this study. We have shown that noisy or poor-quality recordings from a different device can degrade the performance of the classifiers [32]. At the same time, we have shown that the approach (which is identical to the one used in this paper) is particularly well suited to deal with interdevice variations compared with 2 other approaches in the literature [32]. Moreover, the models used in this validation study were trained on audio recorded data through a different audio device than that used in this study, showing further evidence that our approach produces stable results even on other devices. We would also like to emphasize that for the proposed use case and assuming that hospitals provide the technology, hospitals can determine the hardware to be used, which reduces variability between devices and, in turn, improves prediction performance. Third, the cough type and frequency encountered in the data may limit the generalizability of our results, as we performed our validation only in data from patients who were hospitalized with pneumonia overnight. Fourth, our system is a stationary cough monitoring system designed to be placed on a horizontal surface in the vicinity of the patient to be monitored. It does not consider whether the patient leaves the room or carries the smartphone in a pocket or bag. Fifth, we did not address the problem of distinguishing the patient’s cough from the coughs of other people, which is encountered in contact-free audio recordings [43]. Sixth, even though the placement of the smartphones was not standardized, it followed roughly the same guideline for all participants as the smartphones were placed above the bed near the patients (Figure 1). Greater variation in smartphone placement would have strengthened the generalizability of our study. At the same time, there were several sources of variation for which we had no control and could have a greater influence than the position of the smartphone, for example, the general volume of the cough, the exact position of the patient in bed, including head up or down or to the side, people who enter the room and talk. Finally, only 1 female participated in this study. However, the CNN-based model used in this validation study was trained on cough data from a study of 94 patients with asthma, 54 of whom were female. In addition, evaluating the performance of our system using 1 female participant resulted in a slightly improved MCC value of 79%.

### Comparison With Prior Work

Commercially available cough monitoring systems capable of detecting cough in various sensor recordings have been proposed in previous work [21-27]. Some studies have achieved recall and specificity values of >90% [22]. However, these systems operated on coughs recorded under different conditions with different amounts and sensors applied in different contexts, making a comparison with our work difficult. Only a few approaches proposed modes of use comparable with ours in which microphones were not attached to the patient [22,27,31]. According to Barry et al [31], Hull Automatic Cough Counter has a classification performance similar to that of our system. However, the Hull Automatic Cough Counter has not been thoroughly investigated over longer periods and is not supported by an independent validation study. The Cayetano Cough Monitor [27] was validated in a total of 24.5 hours of recordings in another cohort and reported similar classification performance values for detecting cough episodes. Nevertheless, the system performed notably worse than our proposed system in detecting single coughs, with a recall of 51%. Although the Leicester Cough Monitor outperforms our system notably in recall (Table 1), it requires additional operator input for calibration, which takes approximately 5 minutes for 24-hour recordings [44]. Furthermore, none of these systems provide dual functionality to record and detect coughs on devices. To the best of our knowledge, this is the first validation study of a continuous cough monitoring system for smartphones.

Finally, various cough detection approaches involving smartphones and wearable recordings have been proposed and developed in recent literature [18,19,45]. However, they are not commercially available, have not been validated in independent data sets, and have not reported the performance of their systems on devices. Therefore, we have omitted these systems from this discussion.

### Conclusions

In this study, we validated a continuous smartphone-based cough monitoring system for patients with pneumonia in the ward by using 2 execution modes, on device and on computer. Using a commodity device such as a smartphone, our system shows results comparable with the cough monitors available on the market. This research enables scalable and cost-efficient cough monitoring in stationary settings, where the person to be monitored lies flat, for example, in a hospital bed or overnight. Our approach is particularly relevant for chronic diseases such as asthma and chronic obstructive pulmonary disease as well as in the current COVID-19 pandemic, as our proposed system uses smartphones, a contact-free consumer-centric technology that allows convenient, continuous, and remote tracking of coughs.

## Abbreviations

CNN: convolutional neural network
MCC: Matthews correlation coefficient
NPV: negative predictive value
TNR: true negative rate
TPR: true positive rate
